# Case Report: Pheochromocytoma and Synchronous Neuroblastoma in a Family With Hereditary Pheochromocytoma Associated With a MAX Deleterious Variant

**DOI:** 10.3389/fendo.2021.609263

**Published:** 2021-03-17

**Authors:** Diana Borges Duarte, Lia Ferreira, Ana P. Santos, Cláudia Costa, Jorge Lima, Catarina Santos, Mariana Afonso, Manuel R. Teixeira, Rui Carvalho, Maria Helena Cardoso

**Affiliations:** ^1^ Department of Endocrinology, Centro Hospitalar Universitário do Porto (CHUP), Porto, Portugal; ^2^ Department of Endocrinology, Instituto Português de Oncologia Francisco Gentil (IPOFG), Porto, Portugal; ^3^ i3S - Instituto de Investigação e Inovação em Saúde, Universidade do Porto, Porto, Portugal; ^4^ Ipatimup - Institute of Molecular Pathology and Immunology of the University of Porto, Porto, Portugal; ^5^ Faculty of Medicine, University of Porto, Porto, Portugal; ^6^ Department of Genetics, Instituto Português de Oncologia Francisco Gentil (IPOFG), Porto, Portugal; ^7^ Department of Pathology, Instituto Português de Oncologia Francisco Gentil (IPOFG), Porto, Portugal; ^8^ Biomedical Sciences Institute, University of Porto, Porto, Portugal

**Keywords:** MAX gene, pheochromocytoma, hereditary, neuroblastoma, paraganglioma

## Abstract

**Introduction:**

Pheochromocytomas are rare catecholamine-producing neuroendocrine tumours arising from chromaffin cells of the adrenal medulla or extra-adrenal sympathetic paraganglia. Recent studies have indicated that up to 40% of pheochromocytomas could be attributable to an inherited germline variant in an increasing list of susceptibility genes. Germline variants of the MYC-associated factor (*MAX*) gene have been associated with familial pheochromocytomas and paragangliomas with an autosomal dominant pattern of inheritance, a median age at onset of 33 years and an overall frequency estimated at 1.9%. We describe a deleterious *MAX* variant associated with hereditary pheochromocytoma in a family with four affected individuals.

**Case presentation:**

The first patient presented with bilateral pheochromocytoma in 1995; genetic testing was proposed to his oldest son, when he was diagnosed with a bilateral pheochromocytoma with a synchronous neuroblastoma. Upon the identification of the *MAX* variant c.97C>T, p.(Arg33Ter), in the latter individual, his two siblings and their father were tested and the same variant was identified in all of them. Both siblings were subsequently diagnosed with pheochromocytoma (one of them bilateral) and choose to remain on active surveillance before they were submitted to adrenalectomy. All the tumours secreted predominantly norepinephrine, accordingly to the typical biochemical phenotype ascribed to variants in the *MAX* gene.

**Conclusion:**

This case series is, to our knowledge, the one with the largest number of individuals with hereditary pheochromocytoma with a deleterious *MAX* variant in the same family. It is also the first case with a synchronous pheochromocytoma and neuroblastoma in carriers of a *MAX* deleterious variant. This report draws attention to some ill-defined features of pheochromocytoma and other malignancies associated with a *MAX* variant and highlights the importance of understanding the genotype-phenotype correlation in hereditary pheochromocytoma and the impact of oriented genetic testing to detect, survey and treat patients and kindreds at risk.

## Introduction

Pheochromocytomas are rare catecholamine-producing neuroendocrine tumours arising from chromaffin cells of the adrenal medulla (80% to 85% of the cases) or extra-adrenal sympathetic paraganglia (15% to 20% of the cases), the latter also referred as extra-adrenal pheochromocytomas or paragangliomas (1).

In general outpatient clinics, the prevalence of pheochromocytoma in patients with hypertension is 0.1–0.6% (2–4). In recent years there has been an increase in the number of incidentally diagnosed cases, which seems to be related to the greater availability of imaging studies in clinical practice (1). Although epidemiological data on incidence rate is scarce, a recent Dutch study, by Berends et al. (5), found a significant increase in the age standardized incidence rate of pheochromocytomas from 0.29 to 0.46 per 100, 000 person-year between 1995 and 2015.

Recent studies have indicated that up to 40% of pheochromocytomas could be attributable to an inherited germline variant in an increasing list of susceptibility genes (6), which can be grouped into three clusters: pseudohypoxia group (*VHL*, *SDHA*, *SDHB*, *SDHC*, *SDHD*, *SDHAF2*, *FH* and *EPAS1*), kinase signalling group (*RET*, *NF1*, *TMEM127*, *MAX* and *HRAS*) and Wnt signalling group (*CSDE1* and *MAML3*) (7). On this basis, international recommendations suggest that it is essential to offer genetic testing to every patient with a pheochromocytoma as a specific inherited mutation impacts surveillance and monitoring for tumour recurrence, therapeutic approaches, and family screening (7–12).

*MAX* (MYC-associated factor) is a gene associated with regulation of cell proliferation, differentiation, and death (13). Since 2011 (14), germline mutations of the *MAX* gene have been associated with familial pheochromocytomas and paragangliomas with an autosomal dominant pattern of inheritance and a median age at onset of 33 years (range 13-58 years) (7, 11). The overall frequency is estimated at 1.9% and no reliable penetrance estimations are available. The adrenal location is the most common and multifocal tumours are frequent (11, 15). As a kinase signalling pheochromocytoma, it represents a more ultimate cell differentiation with expression of phenylethanolamine N-methyltransferase (PNMT) as the most prominent characteristic, allowing synthesis of epinephrine from norepinephrine (7).

We report a family with hereditary pheochromocytoma carrying a *MAX* deleterious variant in four affected relatives.

## Case report

The patients gave their written consent to sample collection, genetic testing, and the use of genetic test data for the purposes of research. Written informed consent for publication of their clinical details and/or clinical images was obtained from the patients and relative of the patient 2.

### Patient 1 – The Index Case

**Figure 1:**
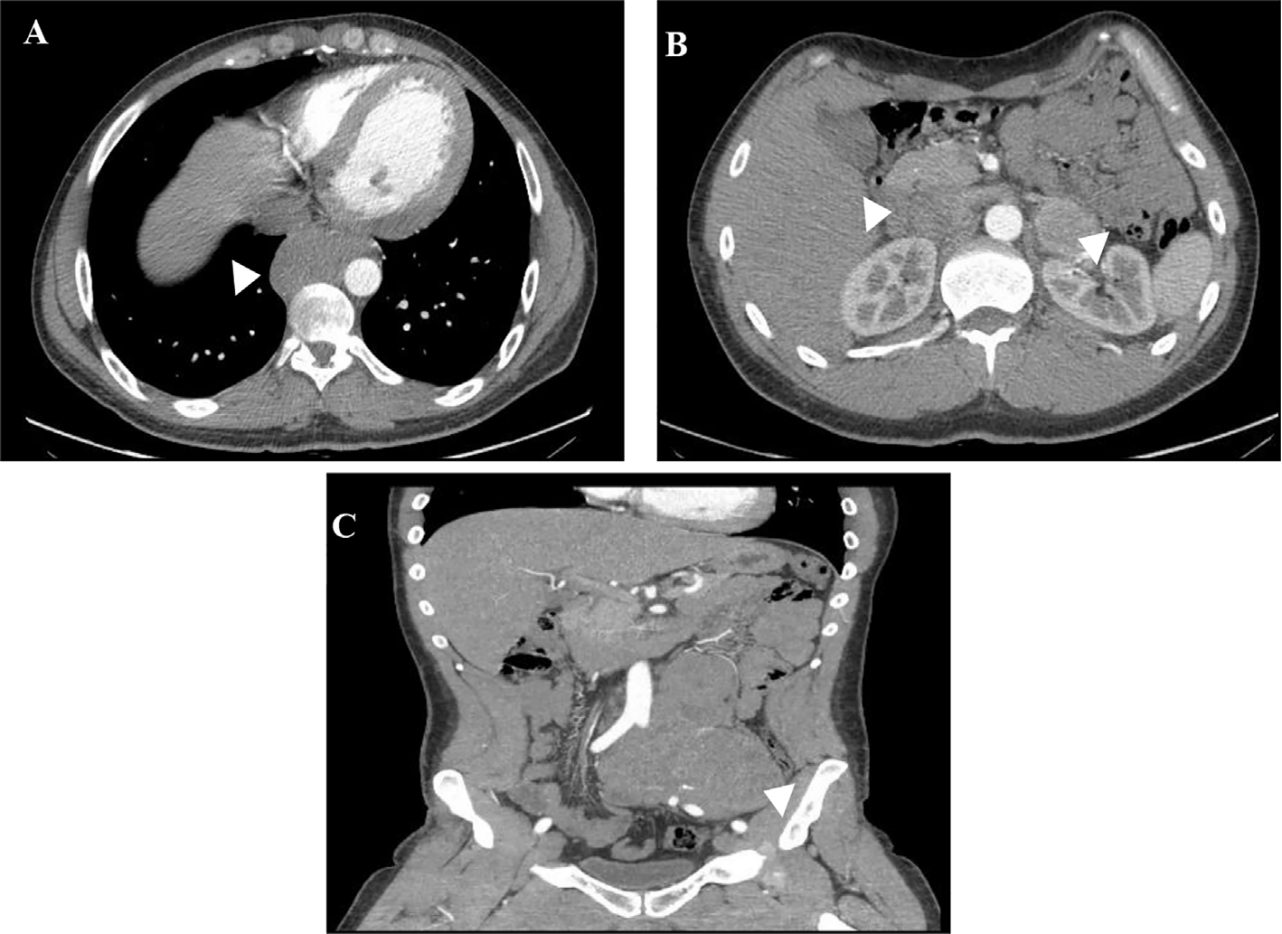
WB-CT scan at diagnosis of patient 1. Posterior mediastinal mass paravertebral to T9-T11 vertebral bodies **(A)**, bilateral adrenal lesions **(B)** and periaortic and peri-common iliac artery lesion with an extensive encasement of this vessel **(C)**.

In November 2010, a 27-year old male, the oldest of the three children of patient 2, was admitted to our hospital for evaluation of an inguinal pain. He had no medical or medication history and was a competitive swimmer. He reported a 3 month-history of inguinal pain, resting tachycardia and a weight loss of 10 kg in the last year that he ascribed to intense exercise and diet. At his first observation on the internal medicine ward, his blood pressure (BP) was 200/100mmHg with a resting heart rate of 140bpm (sinus tachycardia with signs of left ventricular volume overload on EKG) but presented an otherwise normal physical examination. Whole body (WB)-CT scan revealed a posterior mediastinal mass (59x80 mm) paravertebral to T9-T11 vertebral bodies, bilateral hypervascular adrenal lesions (46x39 mm and 15.5 mm on the left and 80x110mm on the right) and a retroperitoneal periaortic and peri-common iliac artery lesion (135x185mm) with an extensive encasement of this vessel with a marked compression of the venous structures (**Figure 1**).

A biopsy of the retroperitoneal mass was performed and was suggestive of a paraganglioma (tumour cells reactive with chromogranin and synaptophysin); for this reason, Endocrinology observation was required. The hormonal workup revealed catecholamine hypersecretion (**Table 1**), ^123^I-metaiodobenzylguanidine (^123^I-MIBG) showed left adrenal and para-aortic and common iliac trace uptake and on echocardiogram there were signs of catecholaminergic cardiomyopathy with severe left ventricular dysfunction (ejection fraction ~20%).

**Table 1:** Urinary catecholamines and metanephrines (reference range) at presentation and during follow-up of Patient 1.

At presentation			Follow-up					
			July 2011	November 2011	August 2012	March 2013	August 2013	December 2013
24-hour urine	Normetanephrines	6506 (480-2424nmol)	1089 (480-2424nmol)	283 (88-444ug)	816 (88-444ug)	1496 (88-444ug)	1067 (88-444ug)	1234 (88-444ug)
	Metanephrines	2618 (264-1729nmol)	78 (264-1729nmol)	<52 (52-341ug)	<52 (52-341ug)	<52 (52-341ug)	<52 (52-341ug)	<52 (52-341ug)

From July’2011 onwards the measurements were performed and kindly provided by the Portuguese Institute of Oncology-Porto.

NM, not measured.

After multidisciplinary review of the case, a metastatic pheochromocytoma and paraganglioma were presumed and surgery was proposed. He needed hospitalization for pre-operative blood pressure and chronotropic control (despite treatment with phenoxybenzamine 100mg, amlodipine 10mg, propranolol 120mg and amiodarone 200mg daily).

He underwent bilateral adrenalectomy with incomplete resection of tumoral mass from the left iliac vessels in February 2011 and pathology was consistent with medullar hyperplasia on the right adrenal gland, and pheochromocytoma with evidence of angioinvasion on the left adrenal gland with tumour cells reactive with chromogranin, synaptophysin and S100. The extra-adrenal tumour was suggestive of well differentiated neuroblastoma (tumour cells reactive with neurofilaments, chromogranin, synaptophysin, NSE and S100) with a Ki-67 index of 10%.

On the immediate post-operative period, his blood pressure and his left ventricular ejection fraction normalised and there was no evidence of catecholamine hypersecretion. ^123^I-MIBG performed on April 2011 showed an intense uptake on his left iliac artery.

Due to significant residual tumoral lesions, the patient was referred to the Portuguese Oncology Institute of Porto (IPOFG, Porto). Next generation sequencing (NGS), using the TruSight Hereditary Cancer Panel (Illumina) identified the *MAX* germline truncating variant c.97C>T, p.(Arg33Ter) (NM_002382.3) in a blood sample. Subsequently, given the unexpected association of neuroblastoma with a variant of the *MAX* gene, the presence of loss of heterozygosity (LOH) was evaluated in a formalin–fixed and paraffin–embedded sample of the neuroblastoma tumour (from a delimited area with >50% tumour cells), using the same NGS panel, and the variant allele frequency (VAF) was 71.2%, which is compatible with LOH. During the follow-up at this centre he presented with recurrence of catecholamine hypersecretion and hypertension. After multidisciplinary team review, it was decided to start palliative chemotherapy for neuroblastoma; despite chemotherapy cycles with cyclophosphamide, doxorubicin, vincristine and topotecan from 2011 to July´2013 there was a disease progression with multiple abdominal and pelvic lymphadenopathies (the largest adenopathic conglomerates with 138mm located by the left iliac vessels and 100x98mm next to renal hilum with bilateral hydronephrosis, severe on the left side). The posterior mediastinal mass’ size remained stable and was deemed unresectable; it was assumed as a probable pheochromocytoma metastasis but with no histological evidence.

On March 2014 he was submitted to exploratory laparotomy with debulking; pathology was consistent with poorly differentiated neuroblastoma with no lymph node parenchyma identified. ^123^I-MIBG on April 2014 documented multiple abdominal and pelvic focus of intense uptake, mainly on the left side. ^131^I- MIBG therapy was performed on June’2014 with no apparent response on post-treatment MIBG scan.

He was afterwards submitted to right nephrostomy and later, on October 2014, to a double-J stent placement, as a preparation for a new surgical debulking. On the 2nd November 2014 he was admitted to the emergency department with an urosepsis with an acute on chronic kidney disease and severe hyperkalaemia. Despite initiation of broad-spectrum antibiotics, vasopressor support and emergent continuous veno-venous hemofiltration, he died 24 hours later, at the age of 31 years old.

### Patient 2

In March 1995, a 32-year old male was referred to our Endocrinology outpatient clinic with complains of daily paroxysms of headaches, diaphoresis, and facial pallor for the last 6 months. A year before he was diagnosed with hypertension, but he had refused anti-hypertensive treatment. At observation he presented a class I obesity (BMI 31.5kg/m^2^), his BP was 156/96mmHg with a resting heart rate of 80bpm and an otherwise normal physical examination. He had no signs of Cushing’s syndrome, such as rounded face, thin skin, easy bruising, or purple striae.

Our investigation revealed an increase in 24-hour urinary normetanephrines and metanephrines (**Table 2**); 1-mg overnight dexamethasone suppression test did not detect autonomous cortisol secretion. Abdominal-CT and MRI revealed bilateral round shaped adrenal lesions of 25 mm with a high signal intensity on T2-weighted images suggestive of pheochromocytoma and the ^123^I-MIBG showed bilateral trace uptake. Phosphocalcic metabolism, calcitonin, thyroid function (and ultrasound) were normal.

**Table 2:** Serum and urinary catecholamines and metanephrines (reference range) at presentation and during follow-up of Patient 2.

At presentation			Follow-up					
			June 1997	June 1998	March 1999	May2000	September 2000	February 2020
24-hour urine	Normetanephrines	1.36 (<1mg)	*NM*	3090 (480-2424nmol)	3305 (480-2424nmol)	*NM*	2134 (480-2424nmol)	*NM*
	Metanephrines	0.54 (<0.4mg)	*NM*	369 (264-1729nmol)	387 (264-1729nmol)	*NM*	69 (264-1729nmol)	*NM*
Serum	Normetanephrines	–	–	–	–	–	–	<100 (<982.8 pmol/L)
	Metanephrines	–	–	–	–	–	–	757 (<456.3 pmol/L)

Laboratory results from the post-operatively period until 1997 were stated on clinical notes but could not be retrieved. Normal catecholamine secretion since the second post-operative period: the immediate post-op (September’2000) and the most recent hormonal workup are shown. Serum metanephrines were not measured at our lab before 2015.

NM, not measured.

In May 1995, a right adrenalectomy and subtotal left adrenalectomy was performed. Pathology revealed adrenal hyperplasia and the genetic testing for RET mutation was negative.

At his immediate post-operative follow-up visit he presented normal blood pressure, but 3 months after surgery he relapsed with recurrence of hypertension and paroxysms associated with elevated urinary metanephrines. Abdominal-CT revealed a residual lesion on the left adrenal gland but ^123^I-MIBG showed no abnormal uptake. After discussing the treatment options with the patient, he opted to resume alpha and beta-block therapy and maintain close clinical, analytical, and imaging surveillance. In June 2000, after clinical worsening with increased frequency of paroxysms associated with retrosternal chest pain, he was submitted to totalization of left adrenalectomy; this time pathology reported a tumoral lesion of 25x15 mm consistent with a pheochromocytoma of low mitotic index. Post-operatively he was started on glucocorticoid and mineralocorticoid hormone replacement and his anti-hypertensive drug was stopped. He has been asymptomatic and with no evidence of catecholamine hypersecretion ever since. After the identification of a deleterious *MAX* variant in the index case (his oldest son, patient 1), he was found to carry the same variant.

### Patient 3

**Figure 2:**
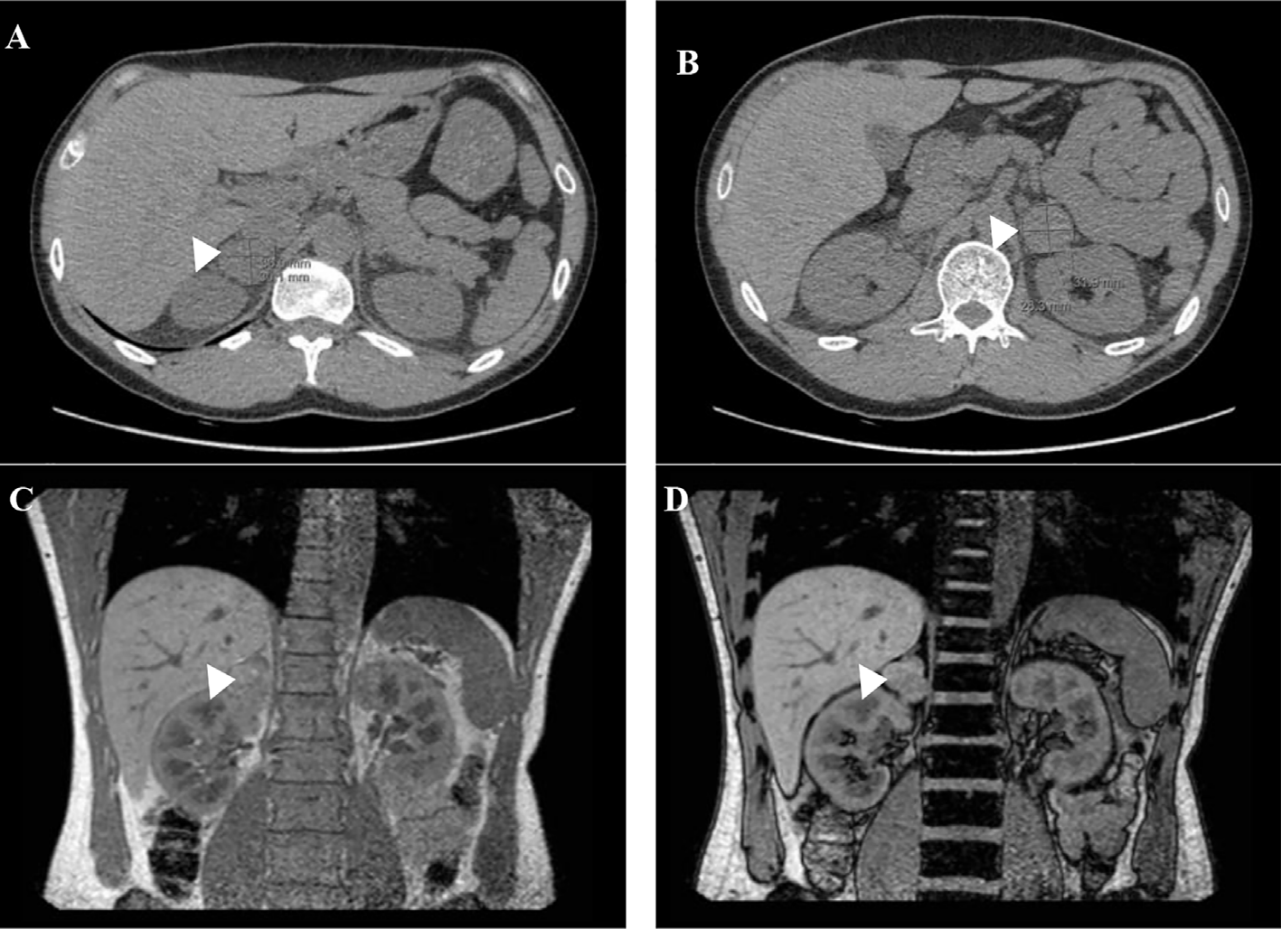
Abdominal-CT **(A, B)** and MRI scan **(C, D)** before bilateral adrenalectomy: adrenal lesions on the right **(A)** and left **(B)** adrenal gland. Coronal in-phase **(C)** and out-of-phase **(D)** MRI images with no loss of signal in the mass.

In 2012, a 26 years old man without significant medical or medication history was brought to our clinic for screening after identification of a *MAX* germline variant in his father (patient 2) and older brother (patient 1). He was overweight (BMI 29.4kg/m^2^) but his physical examination was otherwise normal (blood pressure was 124/74 mmHg and heart rate 90bpm). Biochemical testing revealed a small rise in urinary normetanephrines (see **Table 3**) and the abdominal – CT revealed bilateral adrenal masses (22 mm on the right and 14 mm on the left) both with high density (> 10HU) but without uptake on ^123^I-MIBG. The genetic testing confirmed that he was a carrier of the *MAX* variant previously identified in the family. The patient refused surgery at the time and decided to remain on surveillance. Until 2018 the patient remained asymptomatic when he reports the onset of episodes of palpitations and is documented an elevation of blood pressure (BP 166/87mmHg) and orthostatic tachycardia requiring treatment with a calcium channel blocker, initiated by his primary care physician. Over the years there was a progressive increase in the levels of plasma and urinary normetanephrines (**Table 3**) and in the dimensions of the adrenal lesions (39x27mm on the right and 30x25mm and left adrenal gland on CT scan, both with enhanced contrast on T2 sequences and no signal intensity loss on the opposed-phased image on the MRI **–** **Figure 2**). Echocardiogram had normal ventricular wall thickness with preserved ejection fraction, EKG on sinus rhythm with no signs of ischaemia.

**Table 3:** Serum and 24-hour urinary metanephrines (reference range), by year of patient 3.

	At presentation		Follow-up							
			March 2014	May 2015	April 2016	June 2017	April 2018	May 2019	October 2019	May 2020
24-hour urine	Normetanephrines (480-2424nmol)	3130	3685	6696	11121	*NM*	38243	2976	2002	*NM*
	Metanephrines (264-1729nmol)	1170	1384	1203	1659	*NM*	3839	50	<25	*NM*
Serum	Normetanephrines (< 982.8 pmol/L)	–	–	1390.4	5700	10398	11290	481	302	606
	Metanephrines (< 456.3 pmol/L)	–	–	304.1	548	753	729	<100	<100	<100

At this point the patient accepted surgery and a laparoscopic bilateral adrenalectomy was performed on April’2019 after alpha and beta blockade. Pathology was consistent with bilateral pheochromocytoma with tumour cells reactive with chromogranin, synaptophysin and S100; proliferative index was low (Ki-67<1%).

Post-operatively he was started on glucocorticoid and mineralocorticoid replacement and his anti-hypertensive drug was stopped. Until the last appointment there was no biochemical evidence of catecholamine hypersecretion or relapse of hypertension.

### Patient 4

**Figure 3:**
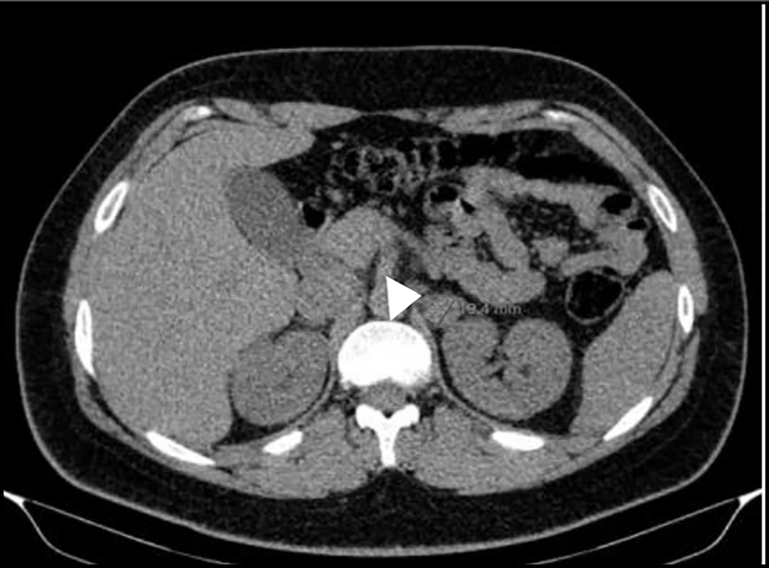
Abdominal-CT scan before left adrenalectomy showing a 17mm nodule on the left adrenal gland.

This is the youngest son of patient 2 and in 2012 he was observed at our clinic after the identification of a *MAX* germline mutation in his father and his older brother. He was 14 years old, had a class I obesity (BMI 31.9kg/m2) with no other medical or medication history nor endocrine hypersecretion features and at examination his blood pressure was 132/67mmHg and heart rate of 85bpm with an otherwise normal physical exam. Genetic testing confirmed that he was a carrier of the same *MAX* variant previously identified in the family. From the age of 15 years old, he underwent annual screening with 24-hour urinary metanephrines and abdominal-CT every two years with both remaining normal until 2017 (**Table 4**) when he presented evidence of norepinephrine hypersecretion and the abdominal-CT revealed on the left adrenal gland, a 17mm nodule with a contrast washout >60% with no other lesions seen on the contralateral gland (**Figure 3**). ^123^I-MIBG scan revealed no abnormal uptake but the ^68^Ga- DOTA-NOC-PET scan documented an uptake on the left adrenal nodule. Like his older brother he chose to remain on active surveillance; although asymptomatic and with normal blood pressure, after considering the unknown risk of malignancy of a deleterious *MAX* variant, he accepted surgery and underwent laparoscopic left adrenalectomy on March 2020 after alpha and beta blockade. Pathology was consistent with pheochromocytoma with tumour cells reactive with chromogranin, synaptophysin and S100; there was also presence of granular cytoplasmatic immunoreactivity for SDHB; proliferative index was low and PASS Score was 0/20.

**Table 4:** Serum and 24-hour urinary metanephrines (reference range), by year of patient 4.

		Follow-up						
		March 2014	April 2016	October 2017	March 2018	March 2019	March 2020	May 2020
24-hour urine	Normetanephrines (480-2424nmol)	1011	1117	6434	3874	*NM*	*NM*	*NM*
	Metanephrines (264-1729nmol)	688	209	1056	586	*NM*	*NM*	*NM*
Serum	Normetanephrines (< 982.8 pmol/L)	–	644	1347	1399	3724	3383	1118
	Metanephrines (<456.3pmol/L)	–	136	<100	110	209	171	141

Serum metanephrines were not measured at our lab before 2015.

NM, not measured.

The immediate post-operative period was uneventful but a month later he reported episodes of headaches with elevated blood pressure and face pallor; serum metanephrines was found slightly elevated. ^123^I-MIBG scan revealed no abnormal uptake.

## Discussion

This case series is, to our knowledge, the one with the largest number of individuals with hereditary pheochromocytoma with a deleterious *MAX* variant in the same family. Although the association between variants in the *MAX* gene and hereditary pheochromocytoma has been established during the past decade, few systematic characteristics are available (16). This report draws attention to some ill-defined features of pheochromocytoma (PPGL) and other malignancies associated with a *MAX* variant.

Firstly, the occurrence of a potential metastatic pheochromocytoma. *MAX* behaves as a classical tumour suppressor gene that encodes for the MAX protein, which interacts with the MYC proto-oncogene and the MAX dimerization protein 1 (MXD1) family of proteins; this MYC/MAX/MXD1 network regulates cell proliferation, differentiation and apoptosis (14, 17). Alterations in this complex promote hereditary susceptibility to neoplasia (18). Previous studies (11, 14, 16, 18, 19) have presented patients with metastatic PPGL but further research is vital to determine the risk of malignancy associated with *MAX* deleterious variants.

In addition to the frequently bilateral PPGL and the early onset, our patients’ tumours secreted predominantly norepinephrine in a greater proportion than epinephrine. This biochemical phenotype is intermediate between the established epinephrine producing tumours due to *NF1* and *RET* variants and the phenotype of norepinephrine tumours harbouring *VHL* or *SDHB*/*D* variants; this diagnostic phenotype is explained by a significant but limited capacity to produce epinephrine due to the intermediate tissue expression of mRNA for PNMT (11). An interesting aspect is the inconsistent uptake of ^123^I-MIBG at the adrenal lesions of our four reported cases. Perhaps different levels of differentiation (and consequently PNMT expression) can be held accountable for this varying accumulation.

Second, the synchronous occurrence of a neuroblastic tumour. The association of PPGL and neuroblastic tumours is uncommon and fewer cases had a genetic link. The majority of these reported cases are composite pheochromocytoma-ganglioneuroma and comprised an association with MEN2 (20–24), VHL (25), NF1 (26–28) and, most recently (29), one case of a new *MAX* gene heterozygous variant, c.299G>C (p.Arg100Pro, NM_002382). Our case is, to the best of our knowledge, the first with a simultaneous PPGL and neuroblastoma presenting as two distinct entities in association with a *MAX* pathogenic variant. LOH observed in the neuroblastoma (VAF=71.2%) supports a causal relation between the *MAX* germline pathogenic variant and the origin of this tumour The embryological common origin and the importance of MYC/MAC/MXD1 network in the development of this neural crest cells tumours are identified as the basis of this association (18). Additionally, evidence suggests that germinative and somatic inactivating *MAX* abnormalities lead to tumour risk, namely renal oncocytoma (18, 19), pituitary adenomas (30–32), pancreatic neuroendocrine tumours (33), small cell lung cancers (34) and Gastrointestinal Stromal Tumours (GIST) (35).

Furthermore, our work highlights the importance of first-degree relatives targeted testing. *MAX* variants present with a parent of origin dependent tumorigenesis and tumour formation occurs almost exclusively through paternal transmission (36). The identification of a germline variant at a PPGL predisposing gene allows the screening of asymptomatic relatives and helps to define a follow-up plan for both mutation carriers and affected individuals (37). Expert recommendations endorse that genetic testing of children is only recommended if they will be offered surveillance during childhood years (11). Although the evidence is limited in *MAX*-related disorders, annual pre-symptomatic biochemical (normetanephrine and metanephrines and methoxytyramine) and biennial imaging (with MRI) surveillance of first-degree relatives (11, 37) should start from five years before the youngest age of onset in the family (11) or no later than 15 years old (38). Patient 4 started his surveillance program by the age of 15 years old but, like his older sibling (patient 3), chose to remain in active surveillance once normetanephrine hypersecretion was confirmed. Due to this mode of inheritance, it is important to analyse the mutation status of patient 3’s son (and future offspring of patient 4) to ascertain who would be at risk.

At last, the extent of surgical procedure, in face of an uncertain PPGL malignancy risk, remains controversial. Regarding our patients, patient 3 postponed his surgery for six years since the diagnosis of bilateral pheochromocytoma; patient 4 also chose to remain on active surveillance until now and was submitted to a unilateral adrenalectomy. Laparoscopic total adrenalectomy has been a standard treatment for unilateral or bilateral adrenalectomy; the main sequel is adrenocortical insufficiency with subsequent need for lifelong mineralocorticoid and corticoid supplementation with risk of both Addisonian crises and excessive steroid replacement (39). Cortical sparing adrenalectomy allows more than 50% of patients to maintain normal adrenal function at 10 years with a PPGL recurrence risk estimated between 0-21%, with even lower reported rates (0-3%) with more recent endoscopic approaches (39–41). Individual risk of malignancy must be taken into account when deciding this surgical procedure. Cortical sparing adrenalectomy is increasingly performed in hereditary PPGL like those with *RET* or *VHL* variants who are associated with a low risk of malignancy and high risk of bilateral PPGL (39). Castinetti (41, 42) reported a low recurrence risk with normal glucocorticoid function in more than 50% of the patients at 10 years. However, this approach is not systematically proposed.

In conclusion, we describe a family with a *MAX* variant associated with hereditary PPGL with one of the patients presenting with a bilateral PPGL with synchronous neuroblastoma, the first case reported to our knowledge. Our case highlights the importance of understanding the genotype-phenotype correlation in hereditary PPGL and the impact of oriented genetic testing to detect, survey and treat patients and kindreds at risk.

## Ethics Statement

Written informed consent was obtained from the individual(s) for the publication of any potentially identifiable images or data included in this article.

## Author Contributions

DB, LF and CC co-wrote the manuscript. MA was the pathologist responsible for the diagnosis of patient 2. JL, CS and MT carried out the genetic analysis that first demonstrated the presence of the MAX gene deleterious variant for patient 2 (in 2011) and the remaining patients (in 2012). RC is the main physician of the patients; APS was the main physician of patient 1 after his referral IPOFG. LF, APS, MT and MC contributed to the revision of the manuscript. All authors contributed to the article and approved the submitted version.

## Conflict of Interest

The authors declare that the research was conducted in the absence of any commercial or financial relationships that could be construed as a potential conflict of interest.
